# Osmolaridade Plasmática nas Doenças Cardiovasculares: Associação, Mecanismos e Significado Clínico

**DOI:** 10.36660/abc.20250694

**Published:** 2026-03-13

**Authors:** Imara Correia de Queiroz Barbosa, Alex Barbosa, Amanda Maíra Damasceno Silva

**Affiliations:** 1 Universidade Federal de Campina Grande Campina Grande PB Brasil Universidade Federal de Campina Grande, Campina Grande, PB – Brasil

**Keywords:** Osmolalidade Plasmática, Sobrecarga de Fluidos, NHANES

As doenças cardiovasculares (DCV) continuam sendo a principal causa de morte no mundo, incluindo no Brasil, onde a doença arterial coronariana (DAC) ocupa o primeiro lugar, seguida pelo acidente vascular cerebral.^1^ Esforços significativos têm sido direcionados para reduzir esse impacto. A busca por novos marcadores de risco cardiovascular tem estimulado intensa pesquisa clínica nos últimos anos.

A osmolalidade plasmática (OSM), um marcador simples do estado de hidratação, reflete o equilíbrio hidroeletrolítico e o estado metabólico do organismo. Pode ser calculada rotineiramente a partir dos níveis de sódio, glicose e ureia, exames amplamente disponíveis na maioria dos sistemas de saúde.^2^ Estudos associaram tanto OSM elevada quanto baixa a pior prognóstico cardiovascular, destacando uma relação complexa em formato de U entre equilíbrio hídrico-eletrólito e desfechos cardiovasculares.^2,3^

Embora todos os componentes da osmolaridade sérica tenham valor prognóstico individualmente em pacientes com DCV, o impacto global da OSM nos desfechos cardiovasculares ainda não é totalmente compreendido. Mais estudos são necessários para esclarecer como esse marcador integrado reflete as complexas interações entre estado de hidratação, distúrbios metabólicos e ativação neuro-hormonal em diferentes contextos clínicos.

Nesta edição dos Arquivos Brasileiros de Cardiologia, Gao et al.^4^ investigaram o papel da OSM como preditor de risco cardiovascular na população geral, utilizando dados de mais de 44.000 participantes do National Health and Nutrition Examination Survey (NHANES). A população do estudo era predominantemente composta por mulheres, brancos não hispânicos e indivíduos com ≤60 anos. No quartil superior (Q4) da OSM os participantes eram mais frequentemente do sexo masculino, mais velhos e apresentavam maior prevalência de hipertensão, diabetes e condições cardiovasculares, incluindo insuficiência cardíaca (IC), angina e infarto prévio. Também apresentavam maior índice de massa corporal, pressão arterial sistólica, ureia e glicose.^4^

Níveis mais elevados de OSM foram associados a maior chance de IC, DAC e infarto do miocárdio, mesmo após ajuste para fatores de risco tradicionais, enquanto a associação com angina desapareceu após ajuste completo. Análises não lineares revelaram relações em formato de U para IC e angina, indicando que tanto valores baixos quanto altos de OSM podem conferir risco. Análises de subgrupos confirmaram essas associações em diferentes faixas etárias, sexos, presença ou ausência de diabetes e categorias de obesidade.^4^

**Figura 1:**
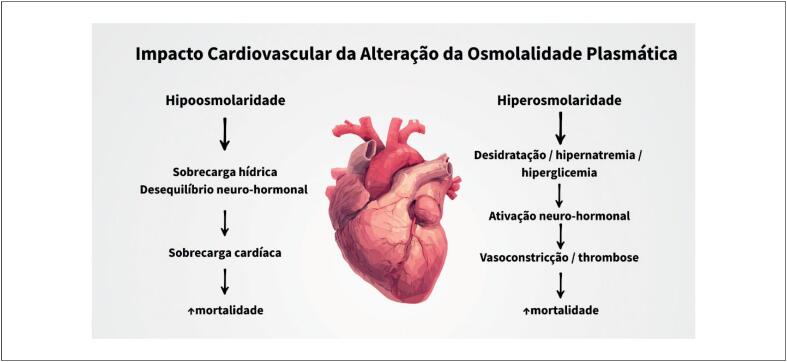
Mecanismos cardiovasculares associados à osmolalidade plasmática alterada.

Esses achados são biologicamente plausíveis. O aumento da OSM frequentemente reflete desidratação, hipernatremia ou hiperglicemia, que podem desencadear ativação neuro-hormonal, vasoconstrição e trombose.^2^ Por outro lado, OSM baixa pode indicar sobrecarga de fluidos, desequilíbrio neuro-hormonal e estresse cardíaco (Figura 1). Juntos, esses mecanismos ajudam a entender como distúrbios sutis no equilíbrio hídrico e eletrolítico podem influenciar desfechos cardiovasculares. Em Kaya et al., a osmolalidade de admissão no quartil mais baixo foi fortemente e independentemente preditiva de mortalidade. Entre os componentes da equação da osmolalidade, o sódio exerce a maior influência nessas alterações, sendo o principal responsável pelo agravamento relacionado à baixa osmolalidade, refletindo o distúrbio eletrolítico mais comum em pacientes com IC hospitalizados, devido à ativação neuro-hormonal ou efeitos de medicamentos.^2^

O estudo apresenta limitações, como seu desenho transversal e dependência de dados autorrelatados para desfechos cardiovasculares, o que pode induzir viés.^4^ Contudo, levanta a importante hipótese de que a OSM, um parâmetro simples e amplamente disponível, poderia, um dia, ser integrada à avaliação de risco cardiovascular. Estudos prospectivos serão essenciais para confirmar esses achados.

Esses resultados estão alinhados com pesquisas anteriores que sugerem que a OSM é um marcador prognóstico em DCV. Hu et al. demonstraram uma associação em formato de U entre OSM e mortalidade por todas as causas em pacientes diabéticos.^3^ De forma semelhante, a razão ureia/albumina (um componente importante da OSM) prediz mortalidade em pacientes com DAC grave e IC.^5^ Importante, em pacientes hospitalizados com IC e fração de ejeção reduzida, a hipoosmolalidade previu de forma independente maior mortalidade de curto prazo, destacando a relevância clínica de ambas as extremidades do espectro de OSM.^6^

Rohla et al. observaram que OSM elevada foi preditiva de mortalidade por todas as causas em pacientes com síndrome coronariana aguda submetidos à intervenção coronária percutânea.^7^

Bhalla et al. avaliaram o significado prognóstico da hiperosmolalidade em pacientes admitidos com acidente vascular cerebral agudo, predominantemente de origem isquêmica. Verificaram que tanto os valores de OSM na admissão quanto os valores máximos, assim como a área sob a curva para medições seriadas, foram maiores em pacientes que morreram em três meses.^8^

Embora esses achados demonstrem uma associação consistente entre OSM e desfechos cardiovasculares, eles podem não representar uma relação causal. Como a OSM é fortemente influenciada por glicose e ureia, seu valor prognóstico pode refletir disfunção metabólica ou renal subjacente, e não a osmolalidade em si.

De forma geral, este estudo contribui para uma melhor compreensão de como medidas bioquímicas simples, como a OSM, podem refinar a estratificação de risco cardiovascular na prática clínica e reforça a necessidade de pesquisas adicionais para determinar se intervenções direcionadas com base na OSM podem melhorar os desfechos.
