# Supplementary material for: Osmolaridade Plasmática nas Doenças Cardiovasculares: Associação, Mecanismos e Significado Clínico
Source: Arq Bras Cardiol. 2026 Mar 13;122(11):e20250694. [Article in Portuguese] doi: 10.36660/abc.20250694 (PMC13155780; doi:10.36660/abc.20250694)
Supplement: Translation

# Translation: Plasma Osmolality in Cardiovascular Disease: Association, Mechanisms, and Clinical Meaning

**DOI:** 10.36660/abc.20250694i

**Authors:** Imara Correia de Queiroz Barbosa, Alex Barbosa, Amanda Maíra Damasceno Silva

**Affiliations:** 1 Campina Grande PB Brazil Universidade Federal de Campina Grande, Campina Grande, PB – Brazil

**Keywords:** Plasma osmolality, Fluid overload, NHANES

Cardiovascular diseases (CVD) remain the leading cause of death worldwide, including in Brazil, where coronary heart disease (CHD) ranks first, followed by stroke.^1^ Significant efforts have been directed toward reducing this burden. The search for new cardiovascular risk markers has fueled extensive clinical research in recent years.

Plasma osmolality (OSM), a simple marker of hydration status, reflects the body's hydroelectrolytic balance and metabolic state. It can be routinely calculated from sodium, glucose, and urea levels, tests widely available in virtually all healthcare systems.^2^ Recent evidence has linked both elevated and low OSM to worse cardiovascular prognosis, highlighting a complex, U-shaped relationship between fluid-electrolyte balance and cardiovascular outcomes.^2,3^

Although all the components of serum osmolality separately have a prognostic value in patients with CVD, the overall impact of OSM on cardiovascular outcomes remains insufficiently understood. Further studies are needed to clarify how this integrated marker reflects the complex interactions between hydration status, metabolic disturbances, and neurohormonal activation in different clinical settings.

In this issue of the Arquivos Brasileiros de Cardiologia, Gao et al.^4^ investigated the role of OSM as a predictor of cardiovascular risk in the general population using data from more than 44,000 participants in the National Health and Nutrition Examination Survey (NHANES) database. The study population was predominantly composed of females, non-Hispanic whites, and individuals aged ≤60 years. Participants in the highest quartile of OSM were more often male, older, and had a higher prevalence of hypertension, diabetes, and cardiovascular conditions, including heart failure (HF), angina pectoris, and prior myocardial infarction. They also presented higher body mass index, systolic blood pressure, blood urea nitrogen, and glucose levels.^4^

Higher OSM levels were associated with increased odds of HF, CHD, and heart attack, even after adjusting for traditional risk factors, while the association with angina disappeared after full adjustment. Nonlinear analyses revealed U-shaped relationships for HF and angina, indicating that both low and high OSM values may confer risk. Subgroup analyses further confirmed these associations across age, sex, diabetes status, and obesity categories.^4^

These findings are biologically plausible. Elevated OSM often reflects dehydration, hypernatremia, or hyperglycemia, which may trigger neurohormonal activation, vasoconstriction, and thrombosis.^2^ Conversely, low OSM can signal fluid overload, neurohormonal imbalance, and cardiac stress (Figure 1). Together, these mechanisms provide insight into how subtle disturbances in water and electrolyte balance may influence cardiovascular outcomes. In Kaya et al., admission osmolality in the lowest quartile was highly and independently predictive of mortality in this patient population. Among the components of the osmolality equation, sodium has the greatest influence on these changes, being the main driver of low osmolality–related worsening, as it reflects the most common electrolyte disorder in hospitalized HF patients due to neurohormonal activation or the effects of medications.^2^

**Figure 1:** Cardiovascular mechanisms associated with altered plasma osmolality.

The study is limited by its cross-sectional design and reliance on self-reported questionnaire data for cardiovascular outcomes, which may introduce bias.^4^ Nonetheless, it raises an important hypothesis that plasma osmolality, a simple and widely available parameter, could one day be integrated into cardiovascular risk assessment. Prospective studies will be essential to confirm these findings.

These results align with prior research suggesting that OSM is a prognostic marker in cardiovascular disease. Hu et al. demonstrated a U-shaped association between plasma osmolality and all-cause mortality in diabetic patients.^3^ Similarly, the ratio of blood urea nitrogen to albumin (a major component of OSM) predicts mortality in patients with severe CHD and HF.^5^ Importantly, in hospitalized patients with HF with reduced ejection fraction, hypo-osmolality independently predicted higher short-term mortality, underscoring the clinical relevance of both ends of the OSM spectrum.^6^

Rohla et al. evaluated the impact of admission OMS on hard clinical endpoints in patients with acute coronary syndrome undergoing percutaneous coronary intervention. They found that higher osmolality was significantly predictive of all-cause death in acute coronary syndrome patients treated with coronary stenting.^7^

Bhalla et al. evaluated the prognostic significance of hyperosmolality in patients admitted with acute stroke, predominantly of ischaemic origin. They observed that both admission and peak osmolality values, as well as the area under the curve for serial measurements, were significantly higher in patients who died within three months compared with survivors. An admission osmolality above 296 mOsm/kg was associated with a higher risk of mortality.^8^

Although these findings demonstrate a consistent association between plasma osmolality and cardiovascular outcomes, they may not represent a causal relationship. Since OSM is strongly influenced by glucose and urea, its prognostic value might reflect underlying metabolic or renal dysfunction rather than osmolality itself.

Overall, this study contributes to a better understanding of how simple biochemical measures, such as plasma osmolality, may refine cardiovascular risk stratification in clinical practice, and underscores the need for further research to determine whether targeted interventions based on OSM can improve outcomes.
